# Carnivorous Plants from Nepenthaceae and Droseraceae as a Source of Secondary Metabolites

**DOI:** 10.3390/molecules28052155

**Published:** 2023-02-24

**Authors:** Magdalena Wójciak, Marcin Feldo, Piotr Stolarczyk, Bartosz J. Płachno

**Affiliations:** 1 Department of Analytical Chemistry, Medical University of Lublin, Chodzki 4a, 20-093 Lublin, Poland; 2 Department of Vascular Surgery and Angiology, Medical University of Lublin, 11 Staszica St., 20-081 Lublin, Poland; 3 Department of Botany, Physiology and Plant Protection, Faculty of Biotechnology and Horticulture, University of Agriculture in Kraków, 29 Listopada 54 Ave., 31-425 Cracow, Poland; 4 Department of Plant Cytology and Embryology, Institute of Botany, Faculty of Biology, Jagiellonian University in Kraków, 9 Gronostajowa St., 30-387 Cracow, Poland

**Keywords:** carnivorous plants, insectivorous plants, secondary metabolites, naphthoquinones, polyphenols

## Abstract

Carnivorous plants are able to attract small animals or protozoa and retain them in their specialized traps. Later, the captured organisms are killed and digested. The nutrients contained in the prey bodies are absorbed by the plants to use for growth and reproduction. These plants produce many secondary metabolites involved in the carnivorous syndrome. The main purpose of this review was to provide an overview of the secondary metabolites in the family Nepenthaceae and Droseraceae, which were studied using modern identification techniques, i.e., high-performance liquid chromatography or ultra-high-performance liquid chromatography with mass spectrometry and nuclear magnetic resonance spectroscopy. After literature screening, there is no doubt that tissues of species from the genera *Nepenthes*, *Drosera,* and *Dionaea* are rich sources of secondary metabolites that can be used in pharmacy and for medical purposes. The main types of the identified compounds include phenolic acids and their derivatives (gallic, protocatechuic, chlorogenic, ferulic, p-coumaric acids, gallic, hydroxybenzoic, vanillic, syringic caffeic acids, and vanillin), flavonoids (myricetin, quercetin, and kaempferol derivatives), including anthocyanins (delphinidin-3-O-glucoside, cyanidin-3-O-glucoside, and cyanidin), naphthoquinones (e.g., plumbagin, droserone, and 5-O-methyl droserone), and volatile organic compounds. Due to the biological activity of most of these substances, the importance of the carnivorous plant as a pharmaceutical crop will increase.

## 1. Introduction

Carnivorous plants are mainly angiosperms, represented by approximately 850 carnivorous plant species [1,2]. Exceptions are some carnivorous liverworts of the genera *Colura* and *Pleurozia* [3,4]. Carnivorous plants are polyphyletic, and carnivorous syndromes have evolved independently in about 10 lineages of flowering plants [5,6,7,8,9]. They are capable of attracting small animals or protozoa and keep them in specialized traps, which are generally of foliar origin [10,11]. However, inflorescences can sometimes be trap organs as well [9]. Captured organisms are killed and digested, and nutrients from their bodies are absorbed by plants to be used for growth and reproduction [1,10,12]. Some researchers propose that carnivorous plant traps are analogous to the animal digestive tract [13].

**Figure 1:**
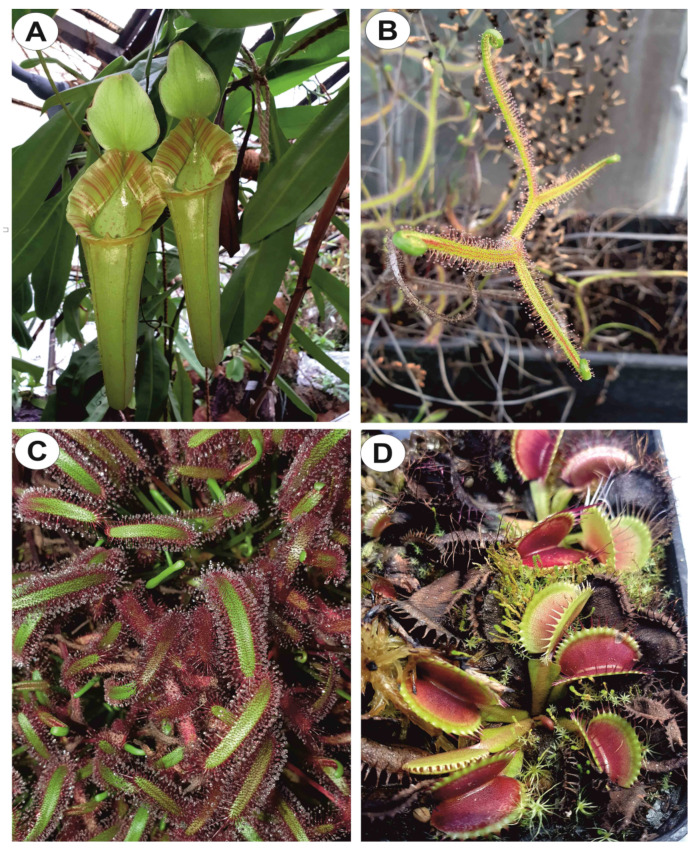
Examples of plants from Nepenthaceae and Droseraceae. (**A**) *Nepenthes* cv. ‘Miranda’, (**B**) Drosera *binata* Labill. var. *dichotoma*, (**C**) Drosera *capensis* L., (**D**) *Dionaea muscipula* J.Ellis.

Carnivorous species are also sources of various secondary metabolites (about 170 compounds), which are used by them to facilitate prey attraction, capture, digestion, and nutrient assimilation as well as defense against pathogens and herbivores or pollinator attraction [14,15]. As reported by Hatcher et al. [15], these metabolites provide a potentially powerful model system for exploring the role of metabolites in plant evolution and in adaptation to extreme nutrient-poor habitats. Moreover, they exhibit diverse biological activity and therefore, potential for use in medicine [14,16,17]. Recently, Miclea provided an overview of secondary metabolites found in carnivorous plants of the Sarraceniaceae family [14]. In our study, we focused on the phytochemistry of Nepenthaceae and Droseraceae species (Figure 1).

The genus *Nepenthes* has a center of diversity in tropical Asia. *Dionaea muscipula* is restricted to a small area of the Atlantic and Gulf Coastal Plain in North America, but the genus *Drosera* is almost cosmopolitan with a large number of species in Australia and South Africa [18]. *Aldrovanda vesiculosa* used to be widespread in the Old World and across various climatic zones; however, now it is rare and endangered [19]. Plants belonging to the Nepenthaceae and Droseraceae families are of great interest to researchers and, in recent years, they have been intensively studied in terms of the biological activity of both crude extracts and isolated components. Furthermore, some species, e.g., *Di. muscipula* and plants from the genus *Drosera* are common in cultivation and easy to propagate and introduce into in vitro cultures. Therefore, their biomass is relatively easily available, which makes these plants a valuable model for biological and phytochemical investigations [20,21,22].

In the review, investigations based on modern identification techniques, such as high-performance liquid chromatography (HPLC) or ultra-high-performance liquid chromatography (UPLC) with mass spectrometry (MS) and nuclear magnetic resonance spectroscopy (MNR) of isolated compounds, have been summarized. The LC-MS-based nontargeted approach was excluded from the study when no compounds were identified. Although it is a powerful tool for the investigation of chemical diversity and useful for the assessment of changes in metabolites in plants for which available data are scarce, it needs further analyses such as nuclear magnetic resonance to elucidate the real structures of the compounds. This approach was applied, for example, to study the ionizable metabolites of pitcher traps and leaf blades in *Nepenthes* × *ventrata* [23], in pitchers of *N. ampullaria*, *N. rafflesiana* and its hybrid (*N.* × *hookeriana*) [24], and in leaves of *Drosera capensis* [25].

The review is based mostly on papers published after 2010, as there are some review papers summarizing older investigations [16,26,27]. However, some oldest articles were also mentioned to give a relevant background or when they provide unique well-documented information. Our paper shows that phenolic acids and their derivatives (gallic, protocatechuic, chlorogenic, ferulic, p-coumaric, gallic, hydroxybenzoic, vanillic, syringic, and caffeic acids, as well as vanillin), flavonoids (myricetin, quercetin, and kaempferol derivatives), including anthocyanins (delphinidin-3-O-glucoside, cyanidin-3-O-glucoside, cyanidin), naphthoquinones (e.g., plumbagin, droserone, 5-O-methyl droserone), and volatile organic compounds, are the main types of compounds investigated in Nepenthaceae and Droseraceae. The role of these compounds in plant physiology, including growth, development, and prey attraction, has been previously described in detail by Hatcher et al. [15].

## 2. Secondary Metabolites in Nepenthaceae and Droseraceae

### 2.1. Phenolic Compounds

Plant phenolics are the most common secondary metabolites in the plant kingdom. This group includes both simple molecules such as phenolic acids and complex compounds with high molecular mass. Phenolics are divided into several classes, i.e., phenols, phenolic acids, coumarins, flavonoids, stilbenes, hydrolyzed and condensed tannins, lignans, and lignins [28,29]. They are synthesized from carbohydrates via the shikimate pathway or via the ‘polyketide’ acetate/malonate pathway during normal plant development and in defense against stress conditions [30].

#### 2.1.1. Phenolic Acids

##### General Information

A characteristic structural feature of phenolic acids is the aromatic ring with one or more hydroxyl substituents and a carboxyl moiety. Two groups of phenolic acids are distinguished: derivatives of benzoic acid and derivatives of cinnamic acid based on the skeleton of C6–C1 and C6–C3, respectively. Phenolic acids can be esterified with alcohols, amino acids, and carbohydrates or may be bonded with malic, tartaric, shikimic, lactic, and quinic acids and may form conjugates with other natural compounds [28,29].

The role of phenolics in plants is varied. They may function as phytoalexins, antifeedants, attractants for pollinators, antioxidants, and protective agents against UV light [28,29,31]. They also have a regulatory function in plant growth and reproduction. Some phenolics, e.g., ferulic and p-coumaric acids, are found in cell walls and contribute to the mechanical strength.

##### Phenolic Acids and Their Derivatives in Nepenthaceae and Droseraceae

As mentioned above, phenolic acids may occur as esters or complex conjugates; thus, acidic or/and alkaline hydrolysis is often performed in the investigation of plant material to release the free form. This approach has been applied by Kováčik et al. [32]. They studied free and bonded phenolic acids in *N. anamensis*, *Drosera capensis*, and *Dionaea muscipula*. HPLC-MS analysis revealed the presence of gallic, protocatechuic, hydroxybenzoic, vanillic, chlorogenic, syringic, caffeic, ferulic, sinapic, and p-coumaric acids and their derivatives in both the leaves and traps of the species. In addition, free salicylic acid was found in *Di. muscipula* and its glycoside- and ester-bound derivatives were present in *Di. muscipula* and *Dr. capensis*. A phenolic aldehyde–vanillin was also found in all species. In turn, a protocatechuic acid derivative (1-O-protochatechoyl glucoside) was found in *Drosera magna* and the structure was established using NMR [33].

The detailed phytochemical profile of polyphenolic constituents, including flavonoids, phenolic acids, and their derivatives, in both field-grown and in vitro propagated leaves of *Dr. rotundifolia* was described by Tienaho et al. The components were tentatively identified based on their retention time, elution order, exact masses, and/or characteristic MS/MS patterns. Ellagic and dimethylellagic acids and their glycosides, as well as quinic acid, monogalloyl glucose, and coumaric acid glycosides were identified in both types of plant material. In turn, methylellagic acid and digalloyl glycose were found only in the field-grown plants [20]. The derivative of ellagic acid-3,3′-di-O-methylellagic acid 4′-glucoside was also identified based on 1H and 13C NMR in aboveground parts of *Dr. tokaiensis* [34]. Furthermore, HPLC-DAD-ESI/MS analysis revealed the occurrence of ellagic acid, the isomer of dimethylellagic acid, 3-O-methylellagic acid, and 3,3′-di-O-methylellagic acid in *Di. muscipula* [35] and 3,3-di-O-methylellagic acid in *Dr. binata* [36].

The presence of gallic, chlorogenic, protocatechuic, ferulic, and p-coumaric acids [22], as well as caffeic, salicylic, and ellagic acids [37] were observed in *Di. muscipula*, and elagic acid was detected in *Dr. binata*, *Dr. indica*, *Dr. spatulata*, and *Di. muscipula* [38]; however, the identification was only based on HPLC separation and comparison with the standards.

**Figure 2:**
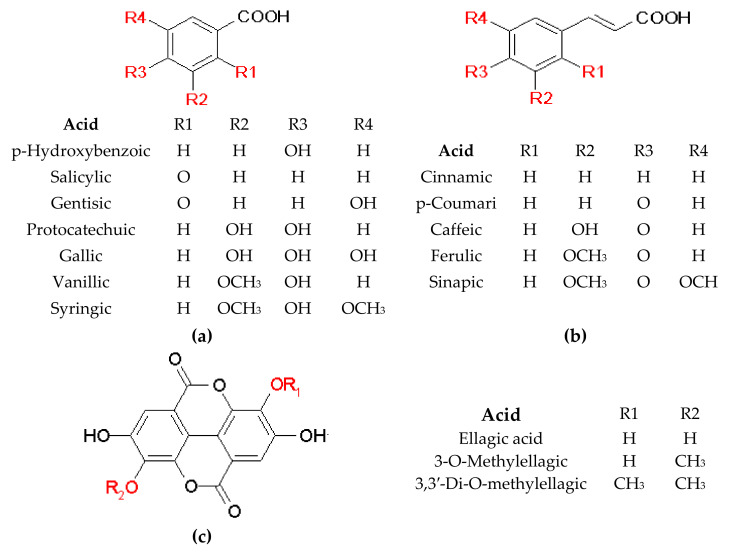
Structures of the most common derivatives of (**a**) benzoic acid, (**b**) cinnamic acid, and (**c**) ellagic acid found in Nepenthaceae and Droseraceae.

Structures of the most common phenolic compounds identified in Nepenthaceae and Droseraceae species are shown in Figure 2.

#### 2.1.2. Flavonoids

##### General Information

**Figure 3:**
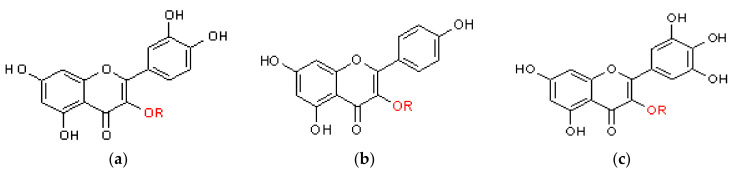
General structure of flavonols (**a**) quercetin, (**b**) kaempferol, and (**c**) myricetin; R—glycoside moiety.

Flavonoids are widespread plant secondary metabolites with a structure based on the C6–C3–C6 backbone. They can be divided into several subclasses: anthocyanins, chalcones, flavanones, flavones, flavonols, isoflavonoids, and flavanols on the basis of their characteristic structural features. They have multiple functions in plant organisms, e.g., protection against the harmful effects of ultraviolet–visible (UV-B) radiation, antimicrobial activity, and an inhibitory effect against herbivores such as insects and nematodes. They are also responsible for yellow-orange coloration and participate in copigmentation with anthocyanins, which increases color intensity and stability [39]. Flavonoids found in Nepenthaceae and Droseraceae plants mostly belong to derivatives of quercetin kaempferol and myricetin (Figure 3).

##### Flavonoids in Nepenthaceae and Droseraceae

The presence of flavonoids in the genera *Nepenthes*, *Drosera*, and *Dionaea* was previously reported in papers from the 1960s to the 1990s. For example, quercetin and its 3-O-galactoside, 3-O-glucoside, and 3-O-digalactoside, gossypetin and its 7-O-glucoside, and 3-O-glucoside kaempferol were detected in *Dr. rotundifolia* [40,41]. Iwashina et al. reported the presence of kaempferol and quercetin 3-O-glycosides in *Di. muscipula* and myricetin and quercetin 3-O-galactosides in *Dr. spathulata* [42]. Moreover, the presence of kaempferol and quercetin glycoside was determined based on the TLC screening of three *Nepenthes* hybrids: *N. rajach*, *N. tentaculata*, and *N. muluensis* [43]. In some studies, identification was based on a comparison of retention times with standards; for example, hyperoside, isoquercitrin, myricetin, quercetin, and ellagic acid were found in *Dr. rotundifolia* and *Dr. madagascariensis* [44], and in *Dr. spatulata* and *Dr. tokaiensis* [45]. However, retention parameters are often misleading, as coelution may occur in the case of compounds with similar polarity, and only further studies based on mass spectrometry and NMR identification can confirm the occurrence of specific derivatives of quercetin and kaempferol. In another study, epicatechin 3-gallate and quercetin gallate esters: quercetin 3-O-(2″-galloylarabinofuranoside), quercetin 3-O-(6″-galloylglucoside), quercetin 3-O-(3″-galloylrhamnoside), quercetin 3-O-(2″-galloylxylopyranoside), and quercetin 3-O-(3″-galloylxylopyranoside), were isolated from *N. gracilis* leaves using preparative chromatography and characterized using C NMR and ESI–MS [46]. The appearance of quercetin and kaempferol after acidic hydrolysis in the trap and leaves of *N. anamensis*, *Dr. capensis*, and *Di. muscipula* was confirmed in HPLC–MS by Kováčik et al. [32], proving the presence of many derivatives of these aglycones in the species. In turn, free flavonoid aglycones: methylated myricetin, quercetin, isorhamnetin, or rhamnetin, kaempferol or fisetin, and their glycosidic conjugates: 3-O-glycosides and 3-O-rhamnosylglucoside kaempferol or fisetin were observed in *Dr. binata* cultured in vitro. As can be seen, some flavonoids were not distinguished based on the collected MS data, because the formula and, therefore, the molecular mass of kaempferol and fisetin, as well as iorhamnetin and rhamnetin are the same (C_15_H_10_O_6_ and C_16_H_12_O_7_, respectively) [47], and more studies are needed to clarify this issue.

Furthermore, NMR and mass spectroscopic data allowed establishing the structure of the main flavonoid, quercetin 3-O-(6′-n-butyl-D-glucuronide isolated from *N. thorellii* × (*ventricosa* × *maxima*) [48]. In turn, Wong et al. conducted a comparative UHPLCQ/TOF-MS-based metabolomic analysis of four *Nepenthes* species, including *N. minima*, *N. ampullaria*, *N. rafflesiana*, and *N. northiana*. They tentatively identified 89 metabolites from different groups and, among others, flavonoid compounds, mostly derivatives of quercetin and kaempferol [49]. In another study, Fourier transform ion cyclotron resonance mass spectra (FT-ICR-MS) and 1H and 13C NMR data allowed establishing the structure of four flavonoids (quercetin, quercetin 3-O-(6′-n-butyl ß-D-glucuronide), quercitrin, kaempferol-3-O-a-L-rhamnoside) isolated from *N. mirabilis* leaves [50], and quercetin, hyperoside, and 2′-O-galloylhyperoside were identified in leaves of *Dr. rotundifolia* based on UHPLC-TOF-MS [51]. In turn, NMR analysis of *Dr. magna* components isolated using column fractionation revealed the presence of tamarixetin-3-rhamnoside, naringenin-6-C-β-D-glucopyranoside, hirsutrin, and four new flavonoid compounds (three flavonol diglycosides and flavan-3-ol glycoside) [33].

Flavonoids identified by MS-MS analysis in *Dr. rotundifolia* grown in the field and propagated in vitro included myricetin glycoside, hyperoside, galloylhyperoside, and hydroxybenzoylhyperin. Additionally, dihydromyricetin, hexahydroxyflavonegalloyl glycoside, tetrahydroxyflavone, kaempferol-galloylglycoside, quercetin, and its derivatives: glycoside and glycoside gallate, were found in plants grown in field conditions. In turn, syringetin glycoside and spinatoside were identified only in in vitro cultivated species [20]. Myricitrine and quercimelin were isolated from the aboveground parts of *Dr. tokaiensis* [34].

##### Anthocyanins

Anthocyanins are plant pigments from the flavonoid class. They are water-soluble compounds that display different colors (red, blue, and purple), and the color depends on pH, light, and temperature [52].

Anthocyanins are found both in reproductive organs (flowers and fruits) and vegetative organs (stems, roots, or leaves). They mostly occur as glycosides and aglycone forms (anthocyanidins) are rarely found in nature. Cyanidin derivatives followed by delphinidin derivatives are the two most common anthocyanins found in plants [52,53]. It should be mentioned that the coloration of plant organs may be a result of the presence of betalains—pigments derived from amino acid tyrosine; however, the presence of betalains and anthocyanins exclude each other [54].

Plant pigments play an important role in the interaction between plants and animals because they attract pollinators and seed dispersers. In carnivorous plants, these pigments are responsible for the distinctive red coloration of the trap, which probably attracts prey; however, the issue is still under debate because many invertebrate species cannot distinguish red wavelengths of light. The other suggested functions of trap pigments include protection against stress related to excess light exposure, nutrient deficiency, ultraviolet radiation, environmental conditions, pathogens, and predators [55].

Although speculation on the presence of anthocyanins in *Nepenthes* species was found in the literature previously, the detection methods used were not selective. A study conducted by Dávila-Lara et al. [56] gave clear evidence. Based on chromatographic parameters, UV–Vis spectra, and mass data obtained with the use of ultra-high performance liquid chromatography-electrospray ionization-high-resolution mass spectrometry (UHPLC–ESI–HRMS), three different cyanidin derivatives, i.e., 3-O-glucoside, 3-O-galactoside, and 3-O-glucuronide, were identified in mature and well-developed pitchers in seven *Nepenthes* species: *N.* × *ventrata* (natural hybrid of *N. ventricosa* and *N. alata*), *N. thorelii*, *N. ventricosa*, *N. robcantleyi*, *N. maxima*, *N. fusca*, and *N. mirabilis.* An analysis of the distribution of anthocyanins in *Nepenthes* × *ventrata* tissue showed that the pigments were most abundant in the peristome, compared to the digestive zone, and 3-O-glucoside was predominant (0.68 vs. ca 0.12 μmol/g of fresh weight, respectively). Low amounts of pigments were also identified in the branches and leaf blades of the plants. Interestingly, the ratio between the anthocyanins was constant in the different tissues [56].

Some studies report the presence of anthocyanins in Droseraceae, including cyanidin and delphinidin 3-O-glucosides in *Di. muscipula* [57], cyanidin and pelargonidin glycosides in *Dr. anglica* and *Dr. intermedia* [58], and cyanidin 3,5-di-O-glucoside, and 3-O-galactoside, as well as pelargonidin 3-O-galactoside and 3-O-glucoside in *Dr. spatulata* [59]. However, it should be noted that the reports were published in 1966–1999 and the investigation was carried out using poorly selective techniques such as column chromatography with spectral analysis or thin layer chromatography (or the methodology was not described); thus, the results need to be verified and the molecular structure should be established with the use of more selective modern techniques.

Only one recent study gives reliable information on anthocyanins in Droseraceae. Henarejos-Escudero et al. investigated pigments in digestive glands, epidermis of the base of the mature stems, and flower petals from *Di. muscipula* using HPLC with mass spectrometry (ESI-MS/MS), and three compounds were found, i.e., delphinidin-3-O-glucoside (myrtillin), cyanidin-3-O-glucoside (kuromanin), and cyanidin. Kuromanin was predominant in the snap trap, whereas myrtillin was the most abundant in mature stems. Aglycone (cyanidin) was found only in the snap trap [60].

**Figure 4:**
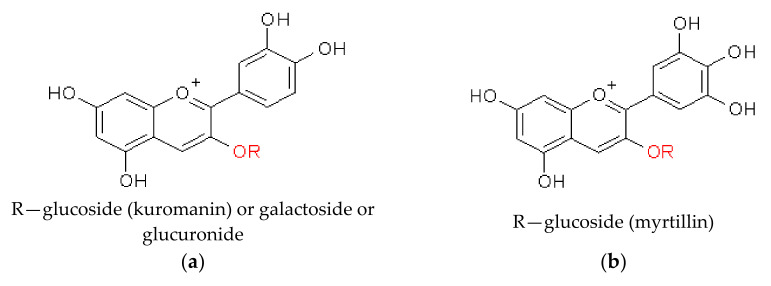
Structure of the most common anthocyanins: (**a**) cyanidin derivatives; (**b**) delphinidin derivatives found in Nepenthaceae and Droseraceae species.

Structures of the most common anthocyanins identified in Nepenthaceae and Droseraceae species are shown in Figure 4.

### 2.2. Naphthoquinones

#### 2.2.1. General Information

Naphthoquinones derived from the naphthalene class are bicyclic structures with two carbonyl groups at positions 1, 4 or 1, 2. They are known toxins and insect ecdysis inhibitors. They also provide antimicrobial protection against visiting preys and preserve the prey during digestion. They show allelopathic effects that might protect plants from pathogen infection and can increase the ability of plants to compete with surrounding organisms for limited resources or to deter herbivores [15,61]. Different substituents may be attached to the ring structure; however, the presence of a hydroxyl and/or methyl group in the quinone structure is typically found in nature.

#### 2.2.2. Naphthoquinones in Nepenthaceae and Droseraceae

Naphthoquinones are characteristic chemotaxonomic markers within Nepenthaceae and Droseraceae families. Several 1,4-naphthoquinone derivatives were found, and plumbagin was the main compound identified. The review paper by Devi et al. listed 114 species [26] in which plumbagin and sometimes other naphthoquinones were detected. Among them, 91 *Nepenthes* species were investigated by Schlauer et al. using TLC and GC-MS analysis of steam distillates and ether extracts from fresh leaves [62]. Furthermore, the studies conducted using GC-MS followed by isolation and elucidation of the structure using 1H-NMR, 13C-NMR have detected plumbagin in *N. khasiana* roots and in the waxy layers at the top prey capture region of the pitchers. In turn, its derivatives, i.e., droserone (3,5-dihydroxy-2-methyl-1,4-naphthoquinone, the oxygenated form of plumbagin) and 5-O-methyl droserone (2-methyl-3-hydroxy-5-methoxy -1,4-naphtho-quinone), were found in pitcher fluid after induction with chitin or prey capture [63,64].

Plumbagin was also detected in the extract from aerial parts of *N.* × *thorelii* (*ventricosa* × *maxima*) [65] and *N.* cv. ‘Miranda’ [66] and in the leaf and pitcher tissue of *N*. × *ventrata*, a natural hybrid of *N. alata* and *N. ventricosa* [67,68]. A detailed study of naphthoquinones in the extract of branches and leaves of *N. mirabilis* conducted by Thanh et al. [50] revealed the presence of droserone, plumbagin, 3-methoxy-7-methyljuglone, 2-methoxy-7-methyljuglone, nepenthone C, and nepenthones F and G.

Although there are some reports on naphthoquinones in *Nepenthes* genus, less is known about this group in Droseraceae family. Only plumbagin was found in *Dr. intermedia* [69] and *D. peltata* var. *lunata* [70], and its structure was established after isolation and NMR analysis. Norman et al. have recently provided more details on the phytochemical profiling of crude extracts from bulbs and leaves of *Dr. magna* followed by isolation and NMR characterization. The study showed the presence of 1,4-naphthoquinones including hydroxydroserone, hydroxydroserone -5-O-β-D-glucoside, droserone, and plumbagin, and two 2,3-dihydronapthalene-1,4-diones [33]. Plumbagin was also determined in *Dr. burmanii* by competitive ELISA using a monoclonal antibody against plumbagin [71], as well as in *Aldrovanda vesiculosa* L. [72] and in *Dr. binata* using HPLC and MS data [47]. In a recent study, 7-methyljuglone (ramentaceone), 7-methyljuglone diglycoside, and 7-methyljuglone glycoside have been found in *Dr. rotundifolia* [20]. Furthermore, plumbagin and 3-chloroplumbagin were identified in *Dr. binata* using HPLC-DAD-ESI/MS [36], plumbagin along with its derivatives (dihydroplumbagin, 3-chloroplumbagin, 8,8′-biplumbagin) were detected in *Di. muscipula* [35], and MS and NMR analysis proved that ramentaceone is a main naphthoquinone in *Dr. aliciae* from in vitro cultures [73,74]. HPLC and a comparison of retention parameters with standards showed the presence of droserone and plumbagin in *Di. muscipula* and *Dr. binata*, droserone and ramentaceone in *Dr. spatulata* and droserone, plumbagin and ramentaceone in *Dr. indica* [38].

Among recent reports, only one paper describes the distribution of naphthoquinones within particular plant organs [64]. Based on the analysis of the *N. khasiana* species, Raj et al. revealed that plumbagin was the most abundant in the roots (approx. 1.4%), followed by the leaf (0.4%) and the stem (0.2%), and only approx. 0.04% was found in the pitcher.

**Figure 5:**
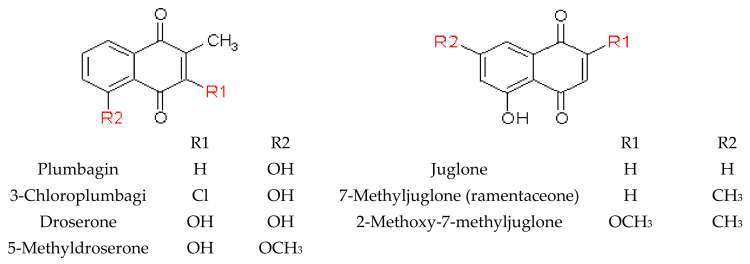
Structure of the most common 1,4-naphthoquinone derivatives identified in Nepenthaceae and Droseraceae.

The structures of naphthoquinones identified in Nepenthaceae and Droseraceae are presented in Figure 5.

#### 2.2.3. Acetogenic Tetralones

**Figure 6:**
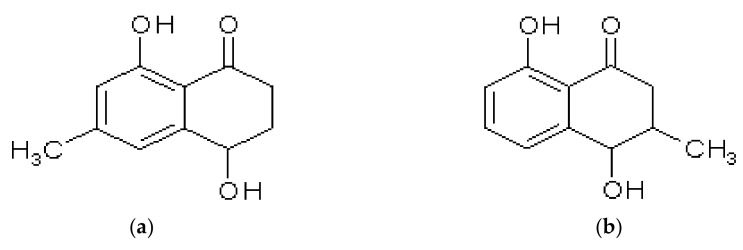
Structure of shinanolone (**a**) and isoshinanolone (**b**).

Acetogenic tetralones are regarded as derivatives of naphthoquinone differing in the presence of the hydroxyl group in position 4 instead of the carbonyl group. A few members of this group, namely isoshinanolone, shinanolone, and epishinanolone (Figure 6), were described by Aung et al. as a component of extract from leaves of *N. gracilis*. After isolation, identification was based on mass and NMR spectra; however, only shinanolone data were shown [75]. Cis-isoshinanolone was found in the extract of *N. mirabilis* branches and leaves [50] and in the aerial parts of *N. thorellii* × (*ventricosa* × *maxima*) [48]. After isolation, the structure was confirmed with the NMR method.

### 2.3. Other Metabolites

Volatile organic compounds (VOCs) involved in the attraction of prey are widely investigated secondary metabolites in carnivorous plants, and many different compounds have been identified. They are lipophilic components with low molecular weight and high vapor pressure at ambient temperature. The composition of VOC is very complex and includes a wide range of compounds from different classes, e.g., volatile terpenes (monoterpenes, diterpenes, and sesquiterpenes), phenylpropanoids, benzenoids, and volatile fatty acids [76]. Gas chromatography with mass spectrometry is the most common method for identification of VOCs. VOCs were identified, for example, in *N. rafflesiana*—fifty-four compounds [77], in *N. rajah*—44 components [78], and in *Di. muscipula*—over 60 VOCs [79]. More details on VOCs in carnivorous plants were provided by Hatcher et al. [25].

Furthermore, a detailed metabolomics analysis based on UHPLCQ/TOF-MS of the aerial parts of *N. minima*, *N. ampullaria*, *N. rafflesiana*, and *N. northiana* revealed the presence of non-phenolic compounds, for example alkaloids [49].

It should also be mentioned that, in addition to secondary metabolites, proteins in pitcher fluid have been intensively studied [80,81,82]. They belong to pathogenesis-related proteins and are responsible for the digestion of prey and the antibacterial effect. For example, 29 proteins belonging to serine carboxypeptidases, α and ß galactosidases, lipid transfer proteins, and esterases/lipases were found to be excreted in *N. mirabilis*, *N. alata*, *N. sanguinea*, *N. bicalcarata*, and *N. albomarginata* pitchers [81].

The secondary metabolites detected in *Nepenthes* and Droseraceae, as well as the methods applied for their determination, are summarized in Table 1, Table 2 and Table 3.

## 3. Biological Potential of Nepentheceae and Droseraceae Species

Carnivorous plants from Nepentheceae and Droseraceae have been used in folk medicine for a long time in the treatment of various disorders [17,27], and recent investigations have confirmed a wide range of activities of both crude extracts and isolated compounds [20,37,51,86].For example, it has been evidenced that *Dr. rotundifolia* and *Dr. tokaiensis* extracts have anti-inflammatory potential [45] and *N. bicalcarata* shows antimicrobial and antidiabetic activity [87]. Antibacterial activity was also evidenced for *N.* cultivar ‘Miranda’ against *Klebsiella pneumonia* [88], for *N. gracilis* against *Bacillus subtilis* and *Escherichia coli* [89], and for *Di. muscipula* against *Staphylococcus aureus*, *Enterococcus faecalis*, *E. coli*, and *Pseudomonas aeruginosa* [21,37]. A biofilm inhibitory effect against *E. coli* strains was also proved for *Dr. rotundifolia* and *Dr. intermedia* [90]. Additionally, *Dr. rotundifolia* extract exhibited antiviral activity and was effective against enteroviruses [20].

Moreover, it has been shown that some species are effective against different types of cancer. *N. adrianii* × *clipeata* showed cytotoxicity toward oral cancer cells [84], *N. thorelii* × (*ventricosa* × *maxima*) exerted anti-leukemic properties [65] and an anticancer effect toward breast cancer [48], and *N.* cultivar ‘Miranda’ was active in the case of lung cancer [88]. It should also be mentioned that the extracts had lower cytotoxicity to normal cells compared to cancer cells.

It is assumed that the majority of the biological properties of Nepenthaceae and Droseraceae species are related to naphthoquinones. It was found that ramentaceone isolated from *Dr. aliciae* [73,74], chloroplumbagin from *Di. muscipula* [91], and plumbagin derived from *N. alata* [92] showed significant anticancer activity. Furthermore, plumbagin (from *N. gracilis*) [93] and its isomer ramentaceone (from *Dr. aliciae*) [86] were shown to be have antibacterial and antifungal properties. Phytochemical screening of anti-inflammatory constituents of *N. mirabilis* also showed that naphthoquinone derivatives were the most potent inhibitors of production of proinflammatory cytokines in cells with lipopolysaccharide-induced inflammation; however, some other constituents, e.g., phenolic compounds, also exhibit an anti-inflammatory effect [94]. Both phenolic compounds and nepenthosides were responsible for the antiosteoporotic activity of *N. mirabilis* extract [50,95] and flavonoids: quercetin and 2″-O-galloylhyperoside were the main components of *Dr. rotundifolia* extract involved in antispasmodic action [51].

## 4. Conclusions and Future Directions

The review showed that species from the genera *Nepenthes*, *Drosera*, and *Dionaea* are rich sources of secondary compounds, which can be grouped into four major types: phenolic acids and derivatives, flavonoids (including anthocyanins), naphthoquinones (including acetogenic tetralones), and volatile organic compounds. Among them, the greatest attention is paid to flavonoids and naphthoquinones, and these metabolites have been intensively studied recently in terms of their biological activity. It has been evidenced that naphthoquinones are cytotoxic against several types of cancer, and they have significant antibacterial, antifungal, antiviral, insecticidal, anti-inflammatory, and antipyretic properties [35,36,94,95,96]. In turn, flavonoids have been shown to exert antiosteoporotic, anti-inflammatory, and antispasmodic effects [50,51,94,95]. This clearly indicates that Nephentaceae and Droseraceae plants have a great potential as a source of components for the development of new drugs and therapies. Furthermore, isolated components may serve as valuable additives to enhance biological effects, for example, antibacterial activity of nanosilver particles [35,36,38].

Obviously, Nephentaceae and Droseraceae are not the only sources of these compounds. For instance, plumbagin is commonly found in members of families Plumbaginaceae and Ebenceae [97]. However, it should be highlighted that many species from the genera *Nepenthes*, *Drosera*, and *Dionaea* can be relatively easily cultivated in vitro and, therefore, they can be a readily available alternative source of metabolites.

The review has shown that there is still scarce information on the chemistry of carnivorous plants including Nepenthaceae and Droseraceae families; in particular, the genus *Aldrovanda* is poorly explored. Therefore, this issue should be intensively studied in the future, especially in the context of reports on the biological activity of extracts or components isolated from some species. The bioactivity-guided approach, which combines the application of modern isolation and identification techniques with assays of the biological activity of particular fractions, seems to be a promising tool for a comprehensive exploration of the biological potential of these species, which may lead to discovering new therapeutic agents [93].

Moreover, future research should be focused on verification of older reports on the phytochemical composition with the use of modern analytical techniques. The investigations should be also extended to other groups of compounds, including primary metabolites, as it has recently been shown that *Dionaea* and *Aldrovanda* traps contain arabinogalactan proteins with potential in industry [98,99], and proteins from *Nepenthes* digestive fluid could help in the therapy of celiac disease [100].

Furthermore, detailed studies on the distribution of particular groups of metabolites in plant tissues should be conducted [101], given the scarcity of such papers shown in this review. Further phytochemical studies should be also focused on the investigation of the numerous cultivars and mutants of *Dionaea* and hybrids of *Nepenthes* because literature data on this topic is scarce so far. It should be noted that the investigation of such plants can lead to a breakthrough in science as has recently been demonstrated by Anda-Larisa et al. [102].

It is also worth increasing the efforts to propagate carnivorous species in vitro and test different elicitors and cultivation conditions, which may significantly increase biomass production and the content of metabolites [103,104].

Our paper summarizes the current knowledge on the occurrence of secondary metabolites in *Nepenthes*, *Drosera*, and *Dionaea*, which can be a good starting point for further investigations and can help researchers dealing with the phytochemistry of these genera.

## Figures and Tables

**Table 1:** Secondary metabolites identified in *Nepenthes*.

Investigated Species	Compound	Method	Ref.
	phenols/phenolic acids and derivatives		
*N. anamensis* (leaf, trap)	gallic: 58.9 (l), 53.7 (t) µg/g; protocatechuic: 4.48 (l), 15.1 (t) µg/g; hydroxybenzoic: 4.28 (l), 9.21 (t) µg/g; vanillic: 2.53 (l), 9.93 (f) µg/g; chlorogenic: 32.4 (l) 53.8 (f) µg/g; syringic: 10.7 (l), 14.7 (f) µg/g; caffeic: 2.6 (l), 3.57 (f) µg/g; ferulic: 1.07 (l), 7.97 (f) µg/g; sinapic: 7.09 (l) 15.7 (f) µg/g; p-coumaric 2.08 (l), 6.77 (t) µg/g; vanillin: 0.85 (l), 0.94 (f) µg/g dw	HPLC-MS	[32]
	flavonoids: flavonols		
*N. minima*, *N. ampullaria*, *N. rafflesiana*, *N. northiana*	quercetin (Q), Q 3-O-rhamnoside, Q 3-(6″-galloylglucoside), Q 3-O-(6″-n-butyl ß-D-glucuronide), rutin, miquelianin, kaempferol (K) 3-glucoside, K 3-O-beta-D-xyloside, 6,8-dihydroxy-K 3-rutinoside, afzelin, luteolin, baicalin, butin, myrciacitrin	UHPLC-Q/TOF–MS	[49]
*N. gracilis* (leaves)	quercetin esters: 3-O-ß-(3″-O-galloylxylopyranoside), 3-O-α-(3″-O-galloylrhamnopyranoside), 3-O-ß-(2″-O-galloylxylopyranoside), 3-O-α-(2″-galloylarabinofuranoside), 3-O-ß-(6″-galloylglucopyranoside)	CC/NMR, MS	[46]
*N. thorellii* × (*ventricosa* × *maxima*) (aerial parts)	quercetin 3-O-(6″-n-butyl-D-glucuronide)	HPLC-DAD, CC/NMR	[48]
*N. mirabilis* (branches and leaves)	quercitrin, quercetin (Q), Q-3-O-b-D-glucuronide, K-3-O-a-L-rhamnoside	CC/NMR, MS	[50]
	flavonoids: anthocyanins *		
*N.* × *ventrata*, *N. thorelii*, *N. ventricosa*, *N. robcantleyi*, *N. maxima*, *N. fusca*, *N. mirabilis* (pitchers: digestive zone—dz, peristome—p)	cyanidin-3-O-glucoside: ca 0.5–6 (p), up to ca 2 (dz) µM/g fw cyanidin-3-O-galactoside: ca 0.1–0.6 (p), up to ca 0.3 (dz) µM/g fw cyanidin-3-O-glucuronide: ca 0–0.1 (p, dz) µM/g fw	UHPLC/HRMS	[56]
	other phenolic compounds		
*N. mirabili* (branches and leaves)*s*	epicatechin	CC/NMR, MS	[50]
*N. gracilis* (leaves)	epicatechin 3-O-gallate	CC/NMR, MS	[46]
*N. minima*, *N. ampullaria*, *N. rafflesiana*, *N. northiana* (aerial parts)	syringin, catechin 5-O-gallate, coniferin, 5-galloylshikimic acid	UHPLC-Q/TOF–MS	[49]
	naphthoquinones		
*N.* cv. ‘Miranda’ (leaves)	plumbagin	GC-MS	[66]
*Nepenthes* × *ventrata* (*N. alata* × *ventricosa*) (pitcher, leaves)	plumbagin	NMR	[67]
*Nepenthes thorellii* × (*ventricosa* × *maxima*) (aerial parts)	plumbagin	HPLC-DAD, CC/NMR	[48]
*N.* × *ventrata* (*N. alata* × *N. ventricosa*) (leaves)	plumbagin	LC-MS/MS	[68]
*N. khasiana* (root, stem, leaves, pitchers),	plumbagin: ca 1.4% (r), 0.2% (s), 0.4% (l), 0.04% (pi) dw * droserone, 5-O-methyl droserone (pi)	GC-MS/NMR	[64]
*N. khasiana* (pitcher liquid)	5-O-methyl droserone, droserone	HPLC/MS, UV, NMR	[63]
*N. mirabilis* (branches and leaves)	3-methoxy-7-methyljuglone2-methoxy-7-methyljuglone, plumbagin, droserone, nepenthones C, F and G	CC/NMR, MS	[50]
*N. alata*, *N. fusca*, *N. gracilis*, *N. mirabilis*, *N. superba*, *N. thorelii*, *N. ventricosa* (pitcher fluids)	plumbagin, 7-methyl-juglone	LC/MS, NMR	[83]
*N. adrianii* × *clipeata* (twigs and leaves)	isoplumbagin	Isolation/NMR	[84]
	acetogenic tetralone		
*N. gracilis* (leaves),	Isoshinanolone, shinanolone epishinanolone	Isolation/MS, NMR	[75]
*N. thorellii* × (*ventricosa* × *maxima*) (aerial parts)	isoshinanolone	HPLC-DAD, NMR	[48]
*N. mirabilis* (branches and leaves)	cis-isoshinanolone	CC/NMR, MS	[50]
	alkaloids		
*N. minima*, *N. ampullaria*, *N. rafflesiana*, *N. northiana* (aerial parts)	trigonelline, anatoxin a, berberastine	UHPLC-Q/TOF–MS	[49]

* values were estimated from the graph; Q—quercetin; K—kaempferol; (dz)—digestive zone; (p)—peristome; (r)—root; (s)—stem; (l)—leaf; (t)—trap; (pi)—pitcher; fw—fresh weight; dw-dried weight.

**Table 2:** Secondary metabolites identified in Drosera.

Species	Compound	Method	Refs.
	phenols/phenolic acids and derivatives		
*Dr. capensis* (leaf, trap)	gallic: 167.8 (l), 195.5 (t) µg/g; protocatechuic: 1.26 (l), 12.1 (t) µg/g; chlorogenic: 28.5 (l), 29.6 (t) µg/g; ferulic: 1.15 (l), 2.21 (t) µg/g; p-coumaric: 1.52 (l), 2.06 (t) µg/g; hydroxybenzoic: 3.72 (l), 17.5 (t) µg/g; vanillic: 1.57 (l) 2.07 (t) µg/g; syringic: 7.86 (l) 10.1 (t) µg/g; caffeic: 1.91 (l) 3.01 (t) µg/g; sinapic: 0.18 (l), 0.69 (t) µg/g; vanillin: 1.93 (l), 1.62 (t) µg/g dw	HPLC-MS	[32]
*Dr. rotundifolia*, *Dr. tokaiensis*, *Dr. spatulata*	ellagic acid (EA)	HPLC	[45]
*Dr. tokaiensis* (aboveground part)	3,3-di-O-methylellagic acid 4′-glucoside	HPLC/NMR	[34]
*Dr. magna* (bulbs and leaves)	1-O-protochatechoyl glucoside	Isolation/NMR	[33]
*Dr. rotundifolia* field (f), in vitro propagated (p)	quinic acid (fp), monogalloyl glucose (fp), digalloyl glucose (f), coumaric acid glycoside (fp), EA (fp), EA glycoside (fp), dimethylEA glycoside ((fp), dimethylEA (fp), methylEA (f)	UPLC/DAD/MS-MS	[20]
*Dr. anglica*, *Dr. intermedia*, *Dr. madagascariensis*, *Dr. rotundifolia* (aerial part)	ellagic acid (0.137–1.107%), 3,3′-di-O-methylEA (0.084–0.143%) dw	LC-MS/NMR	[85]
*Dr. binata* in vitro culture	3,3-di-O-methylellagic acid	HPLC-DAD-MS	[36]
	flavonoids		
*Dr. binata*	methylated myricetin (M) methylated M 3-O-glucoside, quercetin (Q), Q 3-O-glucoside; isorhamnetin (iRh) or rhamnetin (Rh); Rh/isoRh 3-O-glucoside; kaempferol (K)/fisetin (F) K/F 3-O-glucoside; K/F-3-O-rhamnosylglucoside,	HPLC-UV/MS	[47]
*Dr. rotundifolia* field (f), in vitro propagated (p)	myricetin glycoside (fp), hyperoside (fp), galloylhyperoside (fp), hydroxybenzoylhyperin (fp), dihydromyricetin (f), hexahydroxyflavonegalloyl glycoside (f), tetrahydroxyflavone (f), kaempferol-galloylglycoside (f), quercetin (Q)(f), Q-glycoside (f) Q-glycoside gallate (f), syringetin glycoside (p), spinatoside (p)	UPLC/DAD/MS	[20]
*Dr. rotundifolia*	quercetin, hyperoside and 2″-O-galloylhyperoside	UHPLC-TOF-MS	[51]
*Dr. tokaiensis* (aboveground part)	myricitrine, quercimelin	HPLC/NMR	[34]
*Dr. magna* (bulbs and leaves)	tamarixetin-3-rhamnoside, naringenin-6-C-β-D-glucopyranoside, hirsutrin, three new flavonol diglycosides, flavan-3-ol glycoside	Isolation/NMR	[33]
*Dr. rotundifolia Dr. anglica*, *Dr. intermedia*, *Dr. madagascariensis*, (aerial part)	myricetin (M) (<0.003–0.097%), M-3-O-β-galactopyranoside (0.038–0.275%), M-3-O-β-glucopyranoside, hyperoside (H) (0.048–1.530%), 2″-O-galloylH (0–2.515%), isoquercitrin (0.032–0.421%), kaempferol (K), K-3-O-β-galactopyranoside, K-3-O-(2″-O-galloyl)-β-galactopyranoside, astragalin, quercetin (0.056–0.187%) dw	LC-MS/NMR	[85]
	naphthoquinones		
*Dr. binata*, *Dr. adelae*, *Dr. aliciae*, *Dr. capensis*, *Dr. cuneifolia*, *Dr. ramentacea*	plumbagin: 0.001–0.059% fw	HPLC-UV/MS	[47]
*Dr. binata*, *Dr. gigantea*	plumbagin: 2.04 and 0.15 mg/g fw	HPLC-DAD-MS	[35]
*Dr. binata*	plumbagin, 3-chloroplumbagin	HPLC-DAD-MS	[36]
*Dr. intermedia*,	plumbagin	HPLC-MS/NMR	[69]
*Dr. burmanii*	plumbagin	ELISA	[71]
*Dr. peltata Smith* var. *lunata*	plumbagin: 11.05 mg/g dw	TLC/NMR	[70]
*Dr. magna* (bulbs and leaves)	hydroxydroserone (H), H-5-O-β-D-glucoside, droserone, plumbagin, 2,3-dihydronapthalene-1,4-diones	Isolation/NMR	[33]
*Dr. rotundifolia* field (f), in vitro propagated (p)	7-methyljuglone, 7-methyljuglone diglycoside, 7-methyljuglone glycoside (f,p)	UPLC/DAD/MS-MS	[20]
*Dr. aliciae* in vitro culture	ramentaceone	Isolation/NMR, MS	[73,74]

* Q—quercetin; K—kaempferol; M—myricetin; EA—ellagic acid; fw—fresh weight; dw—dried weight; (l)—leaf; (t)—trap.

**Table 3:** Secondary metabolites identified in *Dionaea*.

Species	Compound	Method	Ref.
	phenols/phenolic acids and derivatives		
*Di. muscipula* (leaf, trap)	gallic: 187.8 (l), 167.4 (t) µg/g; protocatechuic: 2.74 (l), 3.21 (t) µg/g; chlorogenic: 24.5 (l), 30.8 (t) µg/g; ferulic: 0.65 (l), 0.69 (t) µg/g; p-coumaric: 0.57 (l), 0.76 (t) µg/g; salicylic: 0.28 (l), 0.19 (t) µg/g; hydroxybenzoic: 4.26 (l), 4.53 (t) µg/g; vanillic: 4.84 (l), 6.87 (t) µg/g; syringic: 8.78 (l), 12.2 (t) µg/g; caffeic: 10.8 (l), 12.0 (t) µg/g; sinapic: 17.0 (l), 42.2 (t) µg/g; vanillin: 2.45 (l), 1.97 (t) µg/g dw	HPLC-MS	[32]
*Di. muscipula*	ellagic, dimethylellagic acid isomer, 3-O-methylellagic acid, 3,3′-di-O-methylellagic acid	HPLC-DAD-MS	[35]
*Di. muscipula*	caffeic: ca 0.18 mg/g, salicylic: ca 300 mg/g, ellagic: ca 0.18 mg/g *	HPLC-DAD	[37]
*Di. muscipula*	chlorogenic:0.26 mg/g, p-coumaric: 0.04 mg/g ferulic: 0.16 mg/g, gallic: 0.31 mg/g, protocatechuic: 0.29 mg/g dw	HPLC-DAD	[22]
	flavonoids: flavonols		
*Di. muscipula*	myricetin: ca 20 mg/g, hyperoside: ca 0.3 mg/g, quercetin: 15 mg/g dw *	HPLC-DAD	[37]
*Di. muscipula*	kaempferol, quercetin after hydrolysis (leaf, trap)	HPLC-MS	[32]
*Di. muscipula*	kaempferol: 0.59 mg/g dw	HPLC-DAD	[22]
*Di. muscipula*	hyperoside, quercetin-3-(6″-O-galloyl)-glucoside/galactoside, kaempferol-3-(6″-O-galloyl)-glucoside	HPLC-DAD-MS	[35]
	flavonoids: anthocyanins		
*Di. muscipula* (digestive glands)	delphinidin-3-O-glucoside, cyanidin-3-O-glucoside, cyanidin	HPLC/MS-MS	[60]
	naphthoquinones		
*Di. muscipula*	plumbagin: 3.45 mg/g fw, dihydroplumbagin, 3-chloroplumbagin, 8,8′-biplumbagin	HPLC/DAD-MS	[35]
*Di. muscipula*	plumbagin: ca 50 mg/g dw *	HPLC-DAD	[37]

* values were estimated from the graph; dw—dried weight; (l)—leaf; (t)—trap.

## Data Availability

Data are contained within the article.
